# Resilience to infection by *Mycobacterium avium* subspecies *paratuberculosis* following direct intestinal inoculation in calves

**DOI:** 10.1186/s13567-018-0553-7

**Published:** 2018-07-13

**Authors:** Kevin J. Stinson, Monica M. Baquero, Brandon L. Plattner

**Affiliations:** Department of Pathobiology, University of Guelph, Guelph, ON Canada

## Abstract

*Mycobacterium avium* subspecies *paratuberculosis* (*Map*) is the cause of Johne’s disease, a chronic enteritis of cattle. A significant knowledge gap is how persistence of *Map* within the intestinal tract after infection contributes to progression of disease. To address this, we exposed calves to *Map* by direct ileocecal Peyer’s patch injection. Our objective was to characterize the persistence of *Map* in tissues, associated intestinal lesions, fecal *Map* shedding, and serum antibody responses, through the first 28-weeks post-inoculation (wpi). Previous work using this model showed 100% rate of *Map* infection in intestine and lymph node by 12 wpi. We hypothesized that direct inoculation of *Map* into the distal small intestine would induce intestinal *Map* infection with local persistence and progression towards clinical disease. However, our data show decreased persistence of *Map* in the distal small intestine and draining lymph nodes. We identified *Map* in multiple sections of distal ileum and draining lymph node of all calves at 4 and 12 wpi, but then we observed reduced *Map* in distal ileum at 20 wpi, and by 28 wpi we found that 50% of animals had no detectable *Map* in intestine or the lymph node. This provides evidence of resilience to *Map* infection following direct intestinal *Map* inoculation. Further work examining the immune responses and host–pathogen interactions associated with this infection model are needed to help elicit the mechanisms underlying resilience to *Map* infection.

## Introduction

Johne’s disease (JD) is a chronic progressive enteric infection of cattle caused by the bacterium *Mycobacterium avium* subspecies *paratuberculosis* (*Map*). Susceptible animals, typically young calves, become infected by ingesting contaminated colostrum, milk, or feces [1]. *Map* invades the intestinal mucosa via M cells overlying Peyer’s patches, where it is taken up by and replicates within resident macrophages. A potent inflammatory response drives recruitment of macrophages and development of granulomatous inflammation at the site of infection [2]. Infected animals then enter a lengthy subclinical period lasting up to 10 years; though these animals often show no clinical signs of infection for several years, they eventually shed *Map* via their feces into the environment and thus perpetuate its spread to susceptible herdmates [2, 3]. Only a small portion of infected animals eventually progress to the invariably fatal clinical disease characterized by malabsorptive diarrhea due to chronic inflammation and thickening of the intestinal mucosa. Diseased animals experience decreased milk production, reduced reproductive efficiency, weight loss and wasting as clinical disease progresses [4]. The mechanisms underlying the early progression of intestinal *Map* infection remain underexplored and largely unknown.

It is difficult to estimate accurately the number of infected herds due to poor sensitivity of screening tests, especially in subclinical animals; however, it is estimated that in North America between 10 and 70% of herds have at least one *Map*-infected animal [5–7]. There is significant economic burden associated with JD of $250 million USD per year in the United States alone, due to loss of productivity and early culling, though these estimates are nearly two decades old [8]. Subclinically diseased animals shed *Map* sporadically and clinically diseased animals shed *Map* progressively; both contribute to environmental contamination and exposure of susceptible calves to contaminated milk, colostrum, and feces [7, 9, 10]. Recent experimental models of calf-to-calf transmission indicated that 50% of contact-exposed animals showed demonstrable infection within 3 months of exposure [11]. Despite routine *Map* exposure, individual animal prevalence in endemic herds ranges between only 5–20% [5, 6]. Thus, a major question exists of why in endemic herds, where susceptible calves are routinely exposed to *Map*, do so few animals ever show evidence of disease? Whittington et al. [12] recently proposed the classification of “resilient” for susceptible animals exposed to *Map*, which show no signs of infection. It remains unclear if such resilience is derived primarily from improved mucosal barrier function, enhanced early pathogen clearance, other factors, or a combination of these factors.

The purpose of this study was to examine the early post *Map*-infection phase within the small intestine of calves to more fully understand persistence of *Map* in intestine and lymph nodes, and development of localized granulomatous lesions following direct experimental inoculation. Use of direct ileal Peyer’s patch inoculation with a measured dose of *Map* allows for further exploration of localized intestinal *Map* infection in calves, an aspect of this disease that remains underexplored. The model has been shown to consistently induce enteric *Map* infection and is unique in its ability to reliably recapitulate the histologic lesions, patterns of fecal *Map* shedding, and immunologic responses consistent with natural subclinical JD [13]. Previous work utilizing this model has only examined the first 12 weeks post-experimental inoculation; thus, new work examining the progression of infection beyond 12 weeks is warranted, to help construct an improved understanding of early enteric *Map* infection.

This model is not intended to investigate the process of *Map* tissue invasion and infection, but instead attempts to recreate an established enteric infection through introduction of a measured dose of *Map* directly into the distal ileum. Studies using mathematical modelling for progression of JD following *Map* infection have shown that antigen load within the intestinal tissues plays a crucial role in driving progression from subclinical to clinical disease [14]. Therefore, our initial hypothesis was that calves directly inoculated with *Map* at the primary site of natural infection would develop persistent localized *Map* infection and then progress towards clinical disease. A more complete understanding of early progression of enteric *Map* infection with this model is likely to have significant impact on understanding and limiting naturally infections.

## Materials and methods

### Bacterial strain and growth conditions

The *Map* strain gc86 was used for inoculation in this study; this is a field strain isolated from the feces of a cow with clinical JD in Ontario, Canada [15]. This *Map* strain has been utilized for a number of JD studies, both in vitro and in vivo [16–19]. Prior to each surgical inoculation, a frozen stock of *Map* gc86 was recovered into Middlebrook 7H9 broth (Becton–Dickinson, Oakville, Ontario, Canada) supplemented with 10% OADC (oleic acid, albumin, dextrose, catalase; Becton–Dickinson Canada), 0.05% Tween 80 (Sigma-Aldrich, Oakville, Ontario, Canada), and 2 mg/L Mycobactin J (Allied Monitor Inc, Fayette, Missouri, USA), and then grown in a stationary flask at 37 °C with 5% CO_2_. Colony forming units were approximated by measuring light absorbance using spectrophotometry at 540 nm and comparison to a previously described standard curve (validated for use with gc86 by confirmation with quantitative culture) [13, 20, 21].

### Inoculum preparation

Following quantification, the *Map* for inoculation of calves was collected by centrifugation of 6 × 10^9^ CFU of *Map* at 1500 × *g* for 20 min. The bacterial pellet was re-suspended in 1.5 mL of sterile phosphate-buffered saline (PBS) to a final concentration of 1 × 10^9^ CFU per 250 µL, and briefly pulse sonicated to disperse bacterial clumps (confirmed via microscopy, and flow cytometry scatter plot). Tuberculin syringes were loaded with 250 µL (representing a total of 10^9^ CFU *Map*) of inoculum and stored at 4 °C until time of surgery (within 12 h of preparation).

Prior to inoculation, the viability of the *Map* contained within inoculum was determined by fluorescein diacetate (Sigma-Aldrich) staining, with analysis by flow cytometry, as previously described [18, 22]. A minimum of 90% viability of *Map* was confirmed prior to animal inoculation.

### Animal trial design

Castrated male Holstein calves between 3 and 4 weeks old were sourced from the University of Guelph Elora dairy research and teaching farm, a farm considered Johne’s disease free based on voluntary participation in a Johne’s disease surveillance program. There have been no cases of clinical Johne’s disease for over 15 years and continuous negative on herd and individual animal level by regular milk and serum ELISA testing. Calves were transported to and housed for the duration of the experiment in an animal biosafety level 2 facility on the main campus of the University of Guelph in Guelph, Ontario. Based upon animal welfare concerns, calves were co-housed in groups of two or four animals per room, with uninfected animals housed separately in dedicated control rooms. Personnel entering the rooms donned clean coveralls, boots, surgical mask, hairnet, and gloves within the antechambers of each animal room. Re-entry into rooms housing control animals was not permitted following contact with *Map*-exposed animals, to eliminate the risk of cross contamination. Animals were maintained on standard non-medicated diets for the duration of the study.

A total of 24 calves were used for this experiment. Calves were randomly assigned into four groups based on pre-determined termination/euthanasia time points and calf availability. Each group contained two control and four *Map*-exposed calves. A 1-week period was given to all calves after arriving into the animal housing facility to allow for environmental acclimation and to ensure health prior to *Map* inoculation. Calves were euthanized by intravenous barbiturate overdose according to their pre-assigned groups, by time following *Map* inoculation: 4, 12, 20, or 28 wpi. All live animal research protocols for this study were pre-approved by the University of Guelph Institutional Animal Care Committee.

### Surgical *Map* inoculation

Calves were inoculated with live *Map* using a surgical inoculation method previously described [13]. Briefly, a 10-cm skin incision was made in the right paralumbar fossa and then extended through the abdominal wall muscle and peritoneum into the abdominal cavity. The distal ileum and cecum were exteriorized, the Peyer’s patches of the distal ileum were visually identified, and then 1 × 10^9^ CFU live *Map* in PBS was injected into the subserosal Peyer’s patch rich region on the anti-mesenteric surface of the distal ileum. Following *Map* injection, 250 µL of sterile diluted India ink was subserosally injected into the distal ileum approximately 5 cm proximal to the site of *Map* injection in order to facilitate localization of the *Map*-inoculation site during tissue collection at post-mortem examination. The distal ileum was then replaced into the abdomen prior to closure of the muscle and skin incisions using standard suture pattern and technique. Calves were not administered antibiotics at any point during the study. A single therapeutic dose (0.5 mg/kg body weight, by intramuscular injection) of meloxicam was administered immediately after surgical *Map*-inoculation and the incision site was treated with topical povidone-iodine to reduce the risk of secondary bacterial infection at the surgical site. No major complications were observed in any calf after surgical inoculation of *Map*. Some animals developed mild localized inflammation at the incision site in the week following surgery; these wounds were treated topically using antiseptic solution until proper healing occurred.

### Feces and blood collection

Feces were collected per rectum prior to *Map* inoculation and bi-weekly thereafter until the end of the study. Feces were immediately stored in 15 mL cryovials at −80 °C until processing. Serum was collected into serum separator tubes via jugular venipuncture prior to *Map*-inoculation and monthly thereafter. Serum was separated from cells and stored at −80 °C until processing.

### Euthanasia, post-mortem examination and tissue collection

Calves were euthanized by intravenous barbiturate overdose, and a thorough post-mortem examination was performed immediately following euthanasia. Four segments of the distal small intestine including ileocecal valve (A), and three sections of ileum proximal to the ileocecal valve at 5 cm intervals (B–D), all of which contained both Peyer’s patch and non-Peyer’s patch regions, and the ileocecal (draining) lymph node were collected. Serial sections of each intestine and lymph node tissue samples described above were preserved in 10% neutral buffered formalin for histologic assessment, snap frozen in OCT compound, and snap frozen in cryovials for storage at −80 °C.

### Histologic lesion scoring

After 24 h immersed in 10% neutral buffered formalin, tissues were trimmed for size, placed into cassettes, dehydrated, and embedded in paraffin. Serial sections were cut at 8 μm and then stained by routine hematoxylin and eosin (H&E) and Ziehl–Neelsen (ZN) according to standard protocols in the Animal Health Laboratory (Lab Services Division, University of Guelph). All tissue sections were visually scored for inflammation as described below, and all ZN-stained sections were scored for the presence or absence of acid-fast bacilli (AFB) by a board-certified veterinary pathologist (BLP), who was blinded to calf ID, experimental *Map* inoculation status and time point after *Map* inoculation of calves for each tissue at the time of scoring.

**Figure 1:**
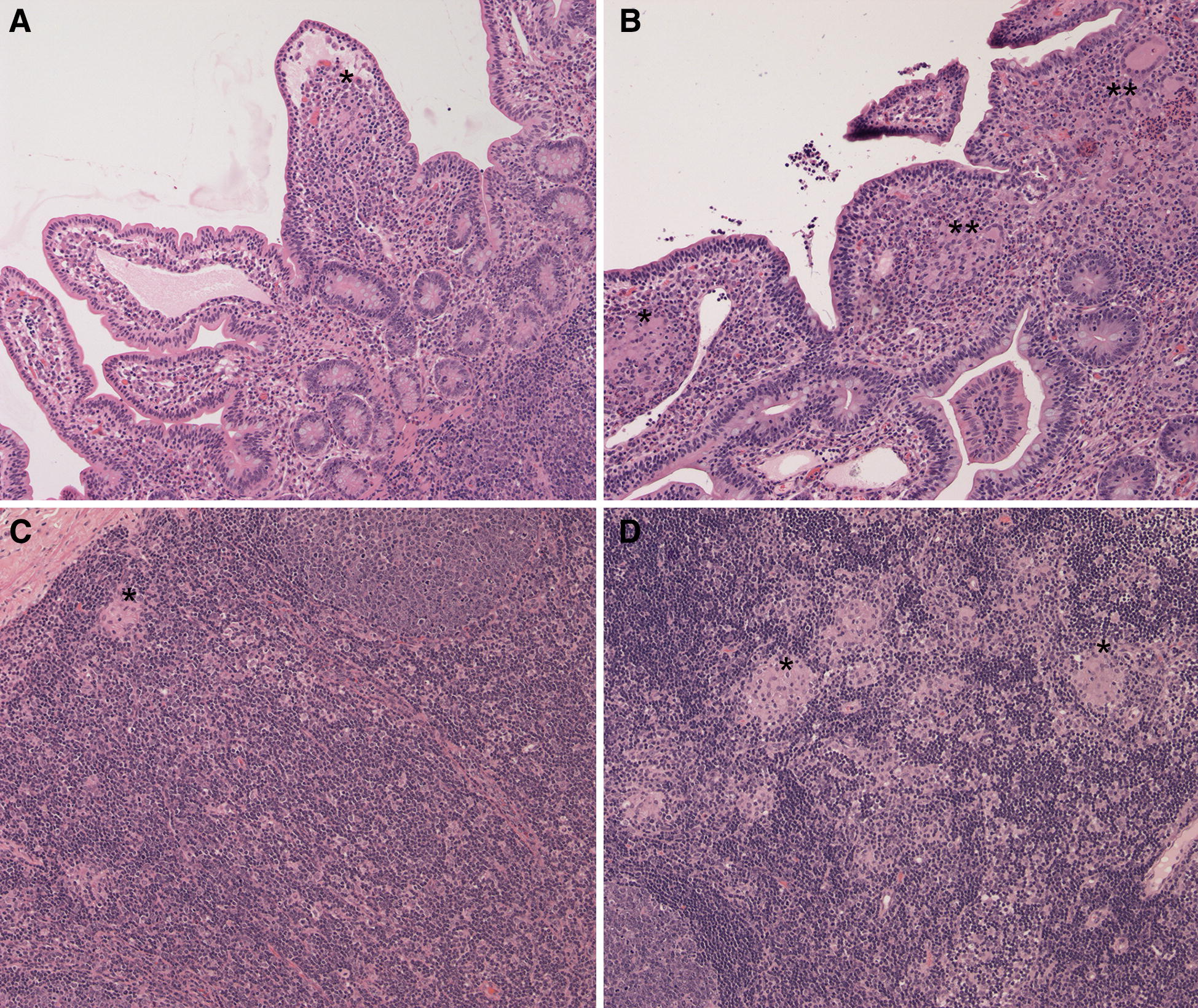
**Histologic lesions identified in small intestine and lymph node.** **A** is ileum, score = 1 due to focal granulomatous inflammation with multinucleated giant cells (*); **B** is ileum, score = 3 due to coalescing granulomatous inflammation in lamina propria (*) and submucosa (not shown), with multinucleated giant cells (**); **C** is lymph node cortex, score = 1, due to focal granulomatous inflammation in the subcapsular cortex (*); and **D** is lymph node cortex, score = 3 due to coalescing granulomatous inflammation in subcapsular cortex (*) and extending throughout the node (not shown). All images are H&E stained, viewed at 100× magnification.

The scoring system for inflammation in the intestine and draining lymph node (Table 1) was modified slightly from several previously published scoring systems in ruminant JD studies [23–25]. Briefly, scores from 0 to 5 were assigned based on the severity and distribution of granulomatous inflammation in each tissue section individually. Scores ranged from a minimum of 0 (no lesions, histologically normal) to a maximum of 5 (severe coalescing to diffuse granulomatous inflammation in the intestinal mucosa or lymph node parenchyma and extending into the submucosa of the small intestine or into afferent or efferent lymphatics of the node). Representative images illustrating the scoring system are shown in Figure 1. Given the range of inflammatory cells in the intestine of normal calves and for the purpose of this study, lesion scores of 0 were considered within normal limits. Scores of 1 were defined as mild lesions, scores of 2–3 were defined as moderate lesions, and scores of 4–5 were defined as severe lesions.

**Table 1:** **Scoring system for identification of histologic lesions by H&E staining**

Score	Definition
0^a^	None
1^b^	Focal granulomatous inflammation (lamina propria or subcortical sinus, cortex only)
2^c^	Multifocal to coalescing granulomatous inflammation (lamina propria or subcortical sinus or cortex only)
3	Coalescing/diffuse granulomatous inflammation (lamina propria, subcortical sinus, cortex)
4^d^	Coalescing/diffuse granulomatous inflammation (extending into submucosa or medulla)
5	Coalescing/diffuse lesions extending deeply into submucosa ± lymphangitis

^a^ Scores of 0 were classified as within normal limits.

^b^ Scores of 1 were classified as mild lesions.

^c^ Scores of 2–3 were classified as moderate lesions.

^d^ Scores of 4–5 were classified as severe lesions.

### Tissue DNA extraction

DNA was extracted from all intestinal and lymph node tissue samples (five samples per calf, tissues A–E as described above) using the DNEasy Blood and Tissue Kit (Qiagen, Toronto, Ontario, Canada), with several modifications. Briefly, 100 mg of frozen tissue per site, compared to 20 mg standard in commercial tissue DNA extraction kits, was digested in 360 μL of buffer ATL and 40 μL of proteinase K overnight at 56 °C with mixing at 1000 RPM on a Thermomixer (Eppendorf Canada, Mississauga, Ontario, Canada). Cell lysis was performed in 400 μL of buffer AL at 95 °C for 5 min. DNA was precipitated with 400 μL of ice cold 100% ethanol at −20 °C for 5 min. The entire sample volume was then loaded onto spin columns in two stages (600 μL at a time), which was followed by two wash steps (each) with buffers AW1 and AW2. DNA was eluted with 50 μL of buffer AE, and stored at −80 °C for downstream processing. Every tissue section which tested negative by PCR (described below), was re-extracted and re-processed, to maximize sensitivity of the diagnostic assay.

### Fecal DNA extraction

Fecal samples for DNA extraction were first subjected to a pre-treatment technique modified from the Mississippi Veterinary Research and Diagnostic Laboratory, with the goal of reducing normal fecal microbiota and increased sensitivity for detection of *Map* [26]. Briefly, 1 g of frozen feces was thawed and diluted in 17.5 mL of pre-lysis buffer (30 mM Tris–HCL pH 8, 0.02N NaOH, 0.1% SDS; final pH 9.65), vortexed for 30 s, and allowed to settle for 30 min. The top 15 mL of supernatant was collected and transferred to a new tube, then pelleted by centrifugation at 1500 × *g* for 20 min. This pellet obtained after pre-treatment was used as the starting point for the DNA extraction using the Stool DNA isolation kit (Norgen Biotek, Thorold, Ontario, Canada). Briefly, DNA was extracted according to the manufacturer’s recommendation, with inclusion of an optional 10-min incubation at 65 °C following pellet resuspension in Lysis Buffer L and Lysis Additive A, prior to bead beating at 30 Hz for 1 min (TissueLyser II, Qiagen, Toronto, Ontario, Canada).

### *Map* PCR

*Map* was detected by PCR using a two-step hemi-nested PCR reaction for the *Map* IS900 element. Accustart PCR Toughmix II (Quanta Biosciences, Beverly, Massachusetts, USA) was used as the PCR master mix, with the addition of 2.5 μg of molecular grade bovine serum albumin. Both rounds of PCR utilized the same cycling conditions, consisting of a 5-min initial denaturation step at 95 °C followed by 35 cycles of: 30 s at 95 °C, 30 s at 68 °C, and 60 s at 72 °C. This was followed by a final 5-min extension at 72 °C. Products were then loaded onto a 1% agarose gel in a Tris–EDTA-Acetic acid buffer, and stained using ethidium bromide. The first round of PCR utilized the forward primer IS900.F1 (5′-CTGGAGTTGATTGCGGCGG-3′), and the conserved reverse primer IS900.R (5′-TGGTTGCGGGGTGGTAGAC-3′) for a 1064 base pair product. The second round of PCR utilized the forward primer IS900.F2 (5′-GATGCGCCACGACTTGCAG-3′) and IS900.R, for an 839 base pair product.

### Validation of molecular assays

PCR primers were designed based on sequence alignment of IS900 and non-*Map* IS900-like elements, available on NCBI GenBank, and targeted to regions conserved in *Map* sequences, but not in non-*Map* sequences. Primers were also selected with a high annealing temperature (68 °C), to reduce the risk of non-specific priming. Together, these factors help reduce the risk of low specificity often attributed to nested PCR methods. The primers were validated against *Map* gc86 using a serial dilution of DNA, with repeatable detection down to 0.025 pg/5 µL, and occasional detection down to 0.0025 pg/5 µL (approximately 5 and 0.5 genomic equivalents respectively, based upon 4 830 000 base pairs). The primers were tested against *Map* K10, and *Mycobacterium smegmatis*, with positive detection of K10 and no detection of *M. smegmatis*. Agarose gel analysis showed one or two bands (two bands present in higher DNA concentrations only), which Sanger sequencing identified as first and second round PCR products. As a comparison, the same serial dilution was run on the Vet Alert commercial *Map* qPCR assay (Tetracore, Rockville, Maryland, USA), with reliable detection to the same lower limit of five genomic equivalents.

To further evaluate the PCR assay, and to validate the tissue DNA extraction, DNA was extracted from two segments of intestinal tissue (ileum) and one segment of lymph node taken from a cow diagnosed with clinical JD (based upon clinical presentation, histopathologic lesions). The hemi-nested PCR assay, combined with the tissue DNA extraction method described here, was able to detect *Map* in these tissues down to a 1/100 000 dilution, while the same dilution series using the Tetracore qPCR kit showed detection only to 1/10 000.

Lastly, fecal DNA extraction was validated by spiking feces with a serial dilution of *Map* gc86, with repeatable detection to a dilution of approximately 5 CFU/g (CFU approximate, determined by spectrophotometry).

### Serum ELISA

*Map*-specific serum antibodies were measured in individual calf sera collected just prior to euthanasia, using a commercially-available *Map* antibody ELISA assay (IDEXX Canada, Markham, Ontario, Canada) at the Animal Health Laboratory, University of Guelph. The IDEXX *Map* antibody ELISA test determines seropositivity of individual samples based on the sample:positive control absorbance ratio (S/P). Samples with an S/P ratio below 0.45 were considered negative, while an S/P ratio above 0.55 was considered positive. An S/P ratio between 0.45 and 0.55 was considered suspect, according to the assay’s guidelines.

### Animal infection status

Animals were defined as exposed, infected, diseased, or resilient based upon clinical case definitions described by Whittington et al. [12]. All animals that underwent surgical *Map* inoculation were considered *Map*-exposed. Calves with positive detection of *Map* in tissues either by PCR or by ZN staining, were considered *Map* infected. Animals with *Map*-induced histologic lesions in ileum and/or lymph nodes were considered diseased. Finally, *Map*-exposed animals but with no *Map* detected in tissues at the time of euthanasia, were considered resilient to *Map* infection. Fecal shedding and presence of *Map*-specific serum antibodies were not included in defining animal infection status, due to the possibility of transient fecal shedding in either unexposed or resilient uninfected animals, or development of antibody responses in resilient animals, as demonstrated in a recent sheep infection study [27].

### Statistical inference

Statistical significance was calculated using R Studio, by generalized linear mixed effect regression modelling using the lme4 package’s “glmer” function. Modelling was designed to examine number of PCR positive tissues by timepoint (logistic regression), number of ZN positive tissues by timepoint (logistic regression), and mean histologic lesion scores by time point (poisson distribution). Animal ID and tissue ID were included as nested random effects, and timepoint as a fixed effect with slope and intercept dependent on animal ID and tissue ID. The general formula for this regression was: FACTOR ~ timepoint + (timepoint|animal/tissue), where FACTOR represents PCR data, ZN data, or histologic lesion scores [28]. Significance was reported as odds ratios with a significance level of α = 0.05. Marginal effects plots were generated using the sjPlot’s “plot_model” function for models which reported significant odd’s ratios.

## Results

### Gross lesions

At post-mortem examination, the location of initial *Map* inoculation site in each calf was confirmed visually based on the presence of India ink. Ink was observed in Peyer’s patches region of the distal ileum 5–8 cm proximal to the ileocecal valve, and frequently in the draining ileocecal lymph node of *Map*-exposed calves. Ileocecal lymph node enlargement was observed in some *Map*-exposed calves; however, lymph node size was variable between calves and not significantly different between *Map*-exposed and control calves. Some calves had fibrous adhesions between the serosal surface of the distal ileum and the omentum or rarely the parietal peritoneum; however, the small intestinal mucosa in all calves was grossly normal and significant gross lesions attributable to *Map* infection were not observed in any animal.

### Histologic lesions and scoring

**Figure 2:**
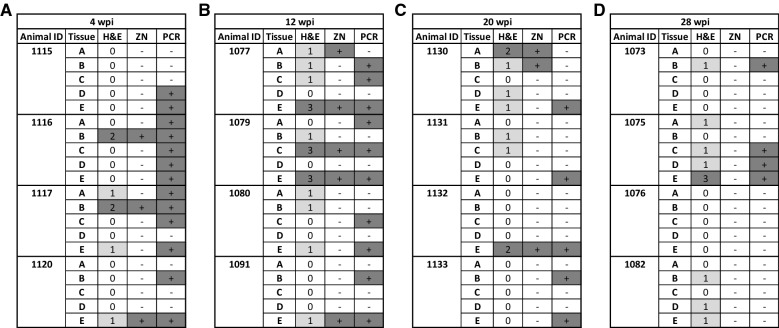
**Lesion scores, ZN, and PCR results for** ***Map*-exposed calves, by weeks post-inoculation (wpi).** Histologic lesions were classified as negative if no lesions were detected, mild (light gray) for lesion scores of 1, or moderate (dark gray) for lesion scores of 2 and 3. No severe histologic lesions were observed. ZN data shows detection of acid fast bacilli (dark gray). Lastly, PCR shows detection of *Map* DNA in tissue by PCR (dark gray). Time points were 4 (**A**), 12 (**B**), 20 (**C**), and 28 (**D**) wpi, examining four ileal tissues (**A**–**D**), and the draining ileocecal lymph node (**E**).

Histologic lesions, lesion scores, and the presence of AFB in intestinal and lymph node segments, respectively, are shown in Figures 1 and 2 with a data summary in Table 2. No calves in control groups (not *Map*-exposed) had evidence of granulomatous inflammation or AFB in any tissues at any time-point in this study. Within the small intestine, granulomatous inflammation attributed to *Map* and AFB were observed predominantly within the lamina propria. The maximum score for granulomatous inflammation in any intestine or lymph node tissue segment in this study was 3.

**Table 2:** **Summary of histologic lesion scores (H&E) and acid fast bacteria (ZN) positive tissues by infection group (timepoint)**

Infection group (wpi)	# mild lesions	# moderate lesions	# severe lesions	Mean lesion score (95% CI)	Maximum lesion score	# ZN positive
4	3	2	0	0.35 (0.05–0.64)	2	3
12	8	3	0	0.85 (0.39–1.31)	3	5
20	5	2	0	0.45 (0.15–0.75)	2	3
28	7	1	0	0.5 (0.17–0.83)	3	0

Experimentally *Map*-inoculated calves in the 4 wpi group had mild (*n* = 3) or moderate (*n* = 2) granulomatous inflammation in 25% (5/20) of all tissue segments. The maximum lesion score observed was 2 (*n* = 2) with a mean lesion score of 0.35 (95% CI of 0.05–0.64). Acid fast bacteria were directly observed following ZN staining in 15% (3/20) of tissue segments.

At 12 wpi, granulomatous inflammation was observed in 55% (11/20) of all tissue segments from *Map*-exposed animals, with eight mild lesions, and three moderate lesions. The maximum lesion score was 3 (*n* = 3), with a mean lesion score of 0.85 (95% CI of 0.39–1.31). Acid fast bacteria were directly observed following ZN staining in 25% (5/20) of tissue segments.

At 20 wpi, granulomatous inflammation was observed in 35% (7/20) of all tissue segments from *Map*-exposed animals, with five mild lesions, and two moderate lesions. The maximum lesion score was 2 (*n* = 2), with a mean lesion score of 0.45 (95% CI 0.15–0.75). Acid fast bacteria were directly observed following ZN staining in 15% (3/20) of tissue segments.

Finally, at 28 wpi, granulomatous inflammation was observed in 40% (8/20) of all tissue segments from *Map*-exposed calves, with seven mild lesions, and one moderate lesion. The maximum lesion score was 3.0 (*n* = 1), with a mean lesion score of 0.5 (95% CI 0.17–0.83). Acid fast bacteria were not directly observed following ZN staining in any of the tissue segments from animals in this time group.

Statistical analysis of tissue inflammation scores and the number of tissues with observed AFB following ZN staining were not significantly different between timepoints. Tissue inflammation scores had an odds ratio of 1.22 (95% CI 0.78–1.91, *P* = 0.386), while ZN positive tissues had an odds ratio of 0.61 (95% CI 0.33–1.14, *P* = 0.120).

### Tissue PCR

*Map* specific tissue PCR data are shown in Figure 2. At all timepoints in this study, no intestine or lymph node tissue segments from control (not *Map*-exposed) calves tested positive for *Map* by PCR.

At 4 wpi, *Map*-exposed animals were positive for *Map* by PCR in 65% (13/20) of intestine and lymph node tissue segments. All calves in the 4 wpi group tested positive for *Map* by PCR in two or more intestinal or lymph node tissue segments. At 12 wpi, *Map*-exposed calves were positive for *Map* by PCR in 50% (10/20) of tissue segments. All *Map*-exposed calves at 12 wpi tested positive for *Map* by PCR in two or more sections of intestine or lymph node. At 20 wpi, *Map*-exposed calves were positive for *Map* by PCR in 25% (5/20) of tissue segments. All calves at this time point tested positive for *Map* by PCR in at least one tissue, and only one animal tested positive in two tissues. Finally, for calves euthanized at 28 wpi, 20% (4/20) of all tissue segments were positive for *Map* by PCR. Only two calves in the 28 wpi group tested positive for *Map* by PCR in at least one tissue segment: one calf tested positive for *Map* by PCR in a single segment of small intestine, and the other calf was positive for *Map* by PCR in two segments of small intestine and the draining ileocecal lymph node. The remaining two *Map*-exposed animals in the 28 wpi group were negative in all tissue segments tested, despite repeated extraction and processing of DNA from all segments of tissue negative for *Map* by PCR, as described above.

**Figure 3:**
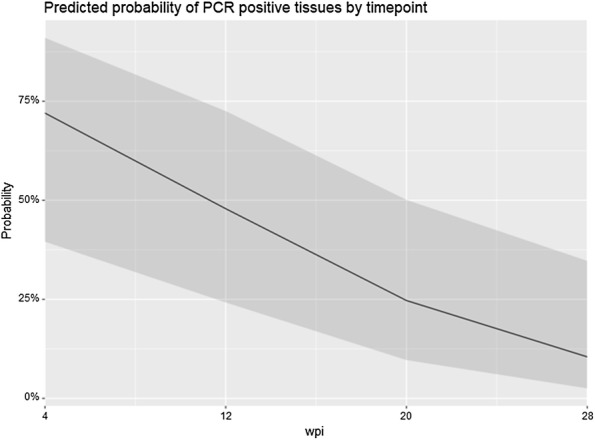
**Marginal effects plot of the PCR statistical model, depicting the probability of PCR positive tissues as a factor of timepoint.** The calculated probability is depicted as the solid line, with the confidence interval depicted as the shaded region. The statistical model identified a significant effect of time on the probability of PCR positive tissues, with an overall odd’s ratio of 0.36 (95% CI 0.18–0.70, *P* = 0.003).

Analysis of *Map* positive tissue segments at each time point identified statistically significant differences between study groups. The logistic model calculated an odds ratio of 0.36 (95% CI 0.18–0.70, *P* = 0.003), suggesting a significant protective effect of time on the probability of *Map* positive tissue segments by PCR (Figure 3).

### Fecal *Map* PCR

**Figure 4:**
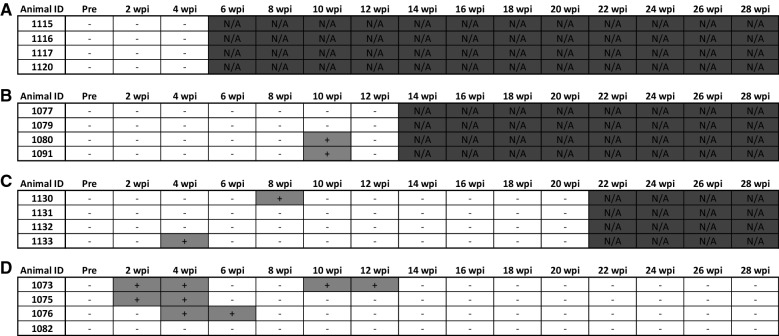
**PCR data for the presence of** ***Map*** **in feces for all** ***Map*-exposed animals by weeks post-inoculation (wpi), including 4 (A), 12 (B), 20 (C), and 28 (D).** Feces collected before inoculation and then at 2-week intervals following inoculation until euthanasia, as described above. Data are shown as positive (+, light grey) or negative (−). Time points not measured are marked N/A and shaded dark grey.

Bi-weekly fecal *Map* PCR data for all animals are shown by time point in Figure 4. Shedding of *Map* in the feces of control calves (not *Map*-exposed) was not detected in this study. *Map* shedding in the feces of *Map*-exposed calves was detected only sporadically throughout this study. As shown in Figure 4A, the 4 wpi group had no detected instances of *Map* shedding in the feces of *Map*-inoculated calves. In the 12 and 20 wpi groups (Figures 4B and C, respectively), *Map* shedding in the feces was detected in 50% (2/4) of the *Map*-exposed animals, but only at two individual time points; intermittent fecal shedding of *Map* in the feces was not detected in these groups. At 28 wpi (Figure 4D), shedding of *Map* in the feces was detected in 75% (3/4) of the animals, and each individual calf shed *Map* in the feces on multiple occasions. In one animal (calf ID 1073), intermittent shedding of *Map* in the feces was detected.

### Serum ELISA

ELISA detecting *Map*-specific serum antibodies at the time of euthanasia in *Map*-exposed calves is shown as S/P ratios in Table 3. Briefly, sera from all calves were negative by ELISA for *Map*-specific antibodies at the termination points of the study, except for a single calf from the 28 wpi group with an S/P ratio of 0.461. This calf was classified as suspect based on the manufacturers recommendation. Control animals all had no detectable *Map*-specific serum antibodies.

**Table 3:** ***Map*** **-specific serum ELISA results**

Infection group (wpi)	Animal ID	S/P ratio	Result
4	1115	0.077	–
	1116	0.029	–
	1117	0.125	–
	1120	0.062	–
12	1077	0.025	–
	1079	0.031	–
	1080	0.045	–
	1091	0.021	–
20	1130	0.06	–
	1131	0.181	–
	1132	0.067	–
	1133	0.061	–
28	1073	0.391	–
	1075	0.461	(+)^a^
	1076	0.086	–
	1082	0.166	–

^a^ Denotes a result of “suspect” by commercial serum ELISA.

### Animal infection status

In the 4, 12, and 20 wpi groups, all *Map*-exposed calves were confirmed to have *Map* infection, based on detection of *Map* by PCR and/or ZN staining in at least one tissue segment. All but one of these calves (1131, 20 wpi) were confirmed to be *Map* infected based on PCR findings in multiple tissues, or PCR findings alongside visual detection of *Map* by ZN staining. However, at 28 wpi, two calves (1076 and 1082) tested negative for *Map* in all tissue segments by both PCR and ZN staining and are thus classified as resilient. One of these calves (1082) had mild sterile granulomatous inflammation in three tissue segments, while the other calf (1076) lacked histologic evidence for *Map*-induced inflammation in all tissues.

## Discussion

It is widely assumed that on endemic *Map*-infected farms the *Map* exposure rate is high, yet only a small number of exposed animals ever show evidence of infection or disease, though experimental evidence has documented a high rate of transmission between exposed and susceptible animals [5, 6, 11]. The question of why *Map* persists and progresses in some individual animals while others do not remains unanswered. The purpose of this study was to examine persistence of *Map* within tissues of calves following direct experimental intestinal inoculation. Based on our previous work, we hypothesized that injection of a large measured dose of *Map* directly into the Peyer’s patches of the distal ileum would induce localized and persistent intestinal *Map* infection, similar to naturally occurring subclinical JD. We also hypothesized that given the route of *Map* exposure the model could be useful for studying the progression of subclinical to clinical JD.

*Map*-exposed animals in this study were examined for evidence of localized *Map* persistence within the tissue using multiple diagnostic tests that are frequently employed for diagnosis of naturally-occurring JD in the field. These data were then used to estimate *Map* persistence, based on both direct (detection of the pathogen by PCR and ZN staining in intestine and draining lymph nodes collected during post-mortem examination) and indirect (patterns of fecal *Map* shedding, granulomatous enteritis, and serological responses) evidence. A major finding in this model was that in the 28 wpi group, 50% (2/4) of the *Map*-exposed animals had no detectable *Map* in the small intestine or draining ileocecal lymph node tissues. Based on the infection status classifications defined above, these animals could reasonably be classified as resilient (no evidence for persistent *Map* tissue infection following direct injection of a large known dose of viable *Map*). The idea of resilience or resistance to *Map* infection is not new and is based on significant experimental and clinical evidence [27, 29–32]. However, the majority of work on the subject of resilience has utilized oral infection models, or naturally-infected animals, where resilience may be at least in part attributable to prevention of *Map* uptake across the mucosal barrier. In the present study, mucosal uptake was bypassed through direct injection of *Map* into the Peyer’s patch rich region of the distal ileum, and this effectively removes mucosal uptake as a factor in understanding persistence in tissue and pathogenesis of early *Map* infection in a calf model. This approach thus adds significant data supporting the idea that with respect to the pathogenesis of the *Map* in calves, resilience to *Map* infection is due, at least in part, to host factors following tissue infection with *Map*.

In our study, calves examined at 28 wpi had a wide variation in the outcome following direct *Map* inoculation. In addition to the two calves classified as resilient, one calf (1075) had evidence for persistent intestinal *Map* infection, with *Map* positively identified by PCR in multiple sections of intestine and the draining lymph node. Based on the definitions of paratuberculosis cases, this calf can be considered subclinically diseased, due to mild granulomatous enteritis attributable to *Map* in three segments of small intestine as well as moderate granulomatous lymphadenitis in the draining ileocecal lymph node [12]. In addition, this particular calf had two instances of fecal *Map* shedding by direct fecal *Map* PCR, as well as evidence of serum *Map*-specific antibodies using a commercial *Map* ELISA test.

Of the two calves classified in this study as resilient, one had mild granulomatous inflammation in two intestinal tissue segments and the draining lymph node, but with no detectable *Map* by PCR or ZN staining. Given the previously reported high success of *Map* infection using direct ileal inoculation (100% at 12 wpi), we believe that this is consistent with residual inflammation due to recovery from intestinal *Map* infection [13]. Unfortunately, one significant limitation to this study is that individual animals were not sampled repeatedly over the time course of the study to properly establish that these animals were truly initially *Map* infected. While this *Map* model has been shown in this and prior studies to reliably induce enteric *Map* infection, classification of an animal as “recovered” requires repeat tissue sampling within the same animal over time, to show evidence of tissue infection at an earlier time point that has subsequently been cleared from the animal [12]. The other calf classified in this study as resilient was histologically normal at 28 wpi, but had two instances of fecal *Map* shedding, at four and six wpi. The fecal shedding patterns observed in this study, particularly in the 28 wpi group, are consistent with findings from other experimental infection studies [11, 27, 32, 33]. However, it is well accepted that fecal shedding can be a transient phenomenon and thus not truly indicative of tissue *Map* infection.

Tissue PCR was the most significant measure of *Map* infection in this study, as statistical analysis identified a significant protective effect of time on the probability of *Map* PCR positive tissues in each infection group. This suggests that in the present infection model, the prevalence of *Map* in tissues decreases over time. Another important finding was the translocation of *Map* from the inoculation site in the distal ileum to the draining ileocecal lymph node. Numerous publications have demonstrated the persistence of *Map* in lymph nodes, suggesting that the draining lymph nodes are a preferred tissue target for improved sensitivity of diagnostic tests during post-mortem examination [34–38]. Given the route of inoculation in this study, there is a possibility that subserosal injection results in increased draining to the local lymph node, and subsequently dissemination outside of the intestinal tract, though site of infection and draining lymph node are likely to remain key sentinel sites for monitoring persistence of *Map* within an animal.

The histologic data in this study were less predictive. Neither the mean histologic lesion scores based on H&E stained tissue segments nor the probability of positive acid-fast bacteria in ZN-stained tissue segments showed any significant differences between groups, as an effect of time. This is likely due to both the low sample size of the study, as well as the generally low mean histologic lesion scores, and number of AFB detected in each infection group.

There is often a question of relevance of the direct inoculation model compared to an oral infection model, and how they relate to natural *Map* infection. Specifically, the subserosal injection utilized in this infection model may show disproportionate delivery of *Map* to the submucosa, which may result in altered host responses, and thus not indicative of natural infection. Our histologic examination of the tissue included examination of the submucosa and subserosa, and we observed lesions attributable to *Map* predominantly within the lamina propria, which is consistent with natural infection. Our data examining *Map* persistence after direct intestinal *Map* inoculation over 7 months are directly comparable to a recent study by Begg et al. where *Map* persistence was examined by intestinal biopsy and then post-mortem examination over a several year period after oral exposure to *Map* [32].

Taken together, this study provides experimental evidence of resilience to *Map* infection following direct inoculation of the bacteria into the distal small intestine, considered the primary site of *Map* infection in natural disease. These data suggest that early localized tissue clearance of *Map* occurs first from the intestinal tract, then from the draining lymph node. The varied outcomes following *Map* exposure in this study show similarities with patterns of *Map*-induced disease observed in the oral model and during natural exposure in endemic herds, but in a much shorter period of time. Consequently, further investigation is required examining host–pathogen interactions in *Map* infection, and how these factors relate to *Map* persistence, and resilience to infection observed within this experimental model.

## Abbreviations

AFB: acid fast bacilli
CFU: colony forming units
H&E: hematoxylin and eosin
JD: Johne’s disease
*Map*: *Mycobacterium avium* subspecies *paratuberculosis*
S/P: sample:positive control ratio
wpi: weeks post-inoculation
ZN: Ziehl–Neelsen
